# Is behavioural flexibility evidence of cognitive complexity? How evolution can inform comparative cognition

**DOI:** 10.1098/rsfs.2016.0121

**Published:** 2017-04-21

**Authors:** Irina Mikhalevich, Russell Powell, Corina Logan

**Affiliations:** 1 School of Mind and Brain, Humboldt-Universitat zu Berlin, Berlin, Germany; 2 Department of Philosophy, Boston University, Boston, MA 02215, USA; 3 Department of Zoology, University of Cambridge, Cambridge, UK

**Keywords:** behavioural flexibility, cognition, convergence, Morgan's canon, simplicity

## Abstract

Behavioural flexibility is often treated as the gold standard of evidence for more sophisticated or complex forms of animal cognition, such as planning, metacognition and mindreading. However, the evidential link between behavioural flexibility and complex cognition has not been explicitly or systematically defended. Such a defence is particularly pressing because observed flexible behaviours can frequently be explained by putatively simpler cognitive mechanisms. This leaves complex cognition hypotheses open to ‘deflationary’ challenges that are accorded greater evidential weight precisely because they offer putatively simpler explanations of equal explanatory power. This paper challenges the blanket preference for simpler explanations, and shows that once this preference is dispensed with, and the full spectrum of evidence—including evolutionary, ecological and phylogenetic data—is accorded its proper weight, an argument in support of the prevailing assumption that behavioural flexibility can serve as evidence for complex cognitive mechanisms may begin to take shape. An adaptive model of cognitive-behavioural evolution is proposed, according to which the existence of convergent trait–environment clusters in phylogenetically disparate lineages may serve as evidence for the same trait–environment clusters in other lineages. This, in turn, could permit inferences of cognitive complexity in cases of experimental underdetermination, thereby placing the common view that behavioural flexibility can serve as evidence for complex cognition on firmer grounds.

## 1. Introduction

According to the standard view in comparative cognition science, animal cognition is generally held to consist in the processes that generate flexible adaptive behaviours in animals [1]. This conception of cognition is motivated by the prima facie plausible assumption that flexible behaviour is underwritten by cognitive processes, and that the more flexible the observed behaviour, the more complex the cognitive processes that underlie it are likely to be (e.g. [2]). This assumption shapes research programmes in comparative cognition, where behavioural flexibility is often treated not only as the gold standard but also as the only significant source of evidence for cognitive complexity [3–5]. Experiments are designed to elicit flexible behaviours that respond appropriately to environmental contingencies, and the observations of such behaviours are, in turn, thought to license inferences about the presence (or absence) of a cluster of cognitive abilities that are generally, if problematically (§2), regarded in the literature as being sophisticated or complex—such as planning, concept formation, metacognition, mindreading and so on [6].

Despite the received view among comparative cognition researchers and philosophers of comparative cognition science that flexible behaviours can serve as evidence of complex cognitive mechanisms, this evidential link has not been explicitly and systematically defended. Such a defence is particularly pressing in the light of the fact that flexible behaviours can often be explained equally well by adverting to putatively simpler cognitive mechanisms. That is to say, the choice between alternative hypotheses that advert to different levels of cognitive complexity is in many cases underdetermined, in that multiple cognitive explanations fit a set of observations equally well with no clear guiding principle for resolving the impasse. As a result, experiments purporting to show results that support an inference of complex cognition remain open to ‘deflationary’ challenges. Moreover, these deflationary hypotheses are often accorded greater epistemic weight because their alternatives are taken to be more complex—and this complexity is thought to warrant a higher burden of proof than that accorded to putatively simpler explanations of equal explanatory power. This methodological state of affairs leaves the evidential connection between flexible behaviour and complex cognition on tenuous grounds.

This paper argues that once behavioural flexibility and cognitive complexity are conceptually disentangled, the preference for simpler explanations is dispensed with, and the full spectrum of evidence (including evolutionary and ecological data) is accorded its proper weight, an argument in support of the prevailing assumption that behavioural flexibility can serve as evidence of complex cognitive mechanisms may begin to take shape. Section 2 explains why behavioural flexibility and cognitive complexity must be conceptually decoupled if the prevailing assumption is to be empirically tenable. Section 3 shows how this conceptual separation leads to a problem of experimental underdetermination—one that is exacerbated by a preference for explanatory simplicity that shapes methodological design in ways that further attenuate the evidential link between behavioural flexibility and forms of cognition that are commonly regarded as complex. Because this preference for simpler explanations appears to be unwarranted on conceptual, theoretical and empirical grounds, it does not adequately resolve the underdetermination problem. Section 4 proposes an adaptive model of cognitive-behavioural evolution, according to which the existence of convergent trait–environment clusters in phylogenetically (and hence developmentally) disparate lineages can serve as evidence for the same trait–environment clusters in other lineages, thus permitting reliable inferences of cognitive complexity in cases of experimental underdetermination. The novelty of this account lays not so much in its theory of the evolution of cognitive complexity *per se*, but in establishing a deeply convergent regularity that can inform hypothesis testing and theory adjudication in experimental psychology settings in which the cognitive capacities of distantly related organisms are investigated. Because the model identifies a non-accidental regularity that is robust across body plans and divergent developmental systems, it can license inferences about the presence of complex cognitive mechanisms in disparate animal groups. This, in turn, can affect the choice of null hypothesis and burden of proof allocation in comparative cognition—a field that traditionally has not drawn on evolutionary concepts, methods and data in designing experiments and interpreting results. The proposed model is then applied to case examples and several objections to its validity are considered.

More broadly, the goal of this paper is to show how evolutionary science can inform experimental programmes that are normally carried out in relative isolation from evolutionary concepts and methods. Evolutionary science is often regarded as a purely historical enterprise, one that is tasked with reconstructing phylogenies and explaining the origins and current distributions of traits by adverting to evolutionary processes that acted on populations in the distant past. This might lead one to think that the epistemic tools and goals of evolutionary biology are orthogonal to psychological investigations of the present cognitive capacities of animals. However, evolutionary concepts and methods, such as those relating to adaptation and homology, can provide clues about what sorts of cognitive capacities may be present in the contemporary time slice of a lineage. They also provide an evolutionary, ecological and phylogenetic context against which to adjudicate alternative proximate cognitive explanations of observed animal behaviour. In illustrating this, the proposed model serves as a corrective for *a priori* methodological biases in comparative cognition, such as the preference for simpler cognitive explanations, which appear to systematically undervalue the evidential weight of behavioural flexibility. Employing a more diverse range of epistemic resources could allow behavioural flexibility to serve (under certain conditions) as reliable evidence of cognitive complexity, thus placing the received view in comparative cognition on firmer footing. Just as importantly, it reveals a rich and largely untapped source of evidence external to the laboratory (in particular, from evolutionary biology) that can be drawn upon to support—or deflate—complex cognition hypotheses.

## 2. Problems of concept and evidence

Although cognition is not typically defined *in terms of* behaviour, it is often equated with the proximate mechanisms that produce flexible behaviour. For instance, as philosopher of cognitive science Kristin Andrews describes it in the Stanford Encyclopedia of Philosophy entry on the subject, ‘animal cognition is constituted by the processes used to generate … flexible behaviour in animal species' [1]. Yet, by building behavioural outputs into the definition of cognition, the link between behavioural flexibility and particular cognitive mechanisms is established by definition. This conceptual coupling is common not only in comparative cognition but also in philosophical action theory (e.g. [7]), where behaviour is often distinguished from mere movement in terms of its proximate cognitive drivers. The problem with this, however, is that such a coupling is only appropriate in cases where the proximate causes of behaviour have already been established *a posteriori*. Initially, observations of some phenomenon P may serve as evidence for the existence of a particular mechanism M that is hypothesized to be a cause of P; as the evidence base for M grows to the point that M is shown to be a cause of P beyond any reasonable doubt, then M will not only come to figure in received explanations of P, but may also become incorporated into the very definition of P. For example, consider the concept of ‘adaptive match’—the functional fit between the traits of an organism and the ecological design problems it needs to solve. Adaptive match was not always understood as the product of natural selection; non-Darwinian evolutionary theories as well as creationist ones were initially offered to explain biological design. As evidence that the mechanism of natural selection was the only plausible cause of adaptive match accumulated and achieved ‘beyond a reasonable doubt’ status in biological science, the concepts of adaptation and function in biology came to build in the mechanism of natural selection. Traits which are now regarded as ‘adaptations’ are those that have been subject to a history of selection for their effects, and these effects are known as their ‘functions’ [8].

In short, it is inadvisable to build specific mechanisms into the definition of a biological trait if that trait is multiply realizable or if its proximate causes are unclear [9]. Although natural selection remains on secure epistemic footing as the only known mechanism for producing complex functional design, the cognitive mechanisms that produce behavioural flexibility are precisely what are at issue in comparative cognition, and there is reason to believe that behavioural flexibility may be multiply realizable (i.e. produced by both sophisticated cognitive mechanisms and putatively simpler ones). Given this enduring controversy in the field of animal cognition, it would clearly be premature to incorporate any particular proximate cognitive mechanisms into the definition of behavioural flexibility or, conversely, to incorporate particular behavioural outputs into the definition of cognition (or complex cognition). Even more problematically, such a coupling prevents observations of behavioural flexibility from *serving as evidence* for the presence of complex cognition.

For these reasons, it is preferable to have a working definition of cognition (and complex cognition) that does not incorporate behavioural outputs. This may seem like an obvious desideratum from the standpoint of biologically oriented approaches to cognition; however, as discussed above, incorporating specific types of behavioural outputs into the definition of cognition or, conversely, delineating certain types of behaviour in terms of the cognitive mechanisms that produce them, is commonplace in comparative cognition and the philosophy of action. There are many definitions of cognition on offer in the literature, which separate cognition from behavioural outputs. For present purposes, we will presume the least controversial and most biologically applicable account among these: namely, the notion that cognition is a form of information-processing and ‘refers to the mechanisms by which animals acquire, process, store and act on information from the environment’ [10, p. 4].

This ‘big tent’ approach to cognition grants minimal forms of cognition to a wide variety of organisms with very different neurological capacities, sensory modalities, and lifeways (for defences of such an account, see [11,12]). At the same time, it rejects narrow ‘top-down’ approaches that restrict cognition to a small range of organisms by presuming that cognition entails some highly atypical property, such as the possession of language or reason. Restrictive accounts of cognition are generally built around the intuition that some entities (e.g. microbes, plants, termites, cruise missiles, etc.) are paradigmatically non-cognitive while others (e.g. average adult humans) are paradigmatically cognitive, with the success of a given account gauged by how well it conforms to these intuitions. For instance, after honeybees were discovered to be capable of marked and rapid learning, the criterion of learning as a mark of intelligence was discarded. As Chittka *et al*. [13, p. 2678] note, ‘there may be good reasons not to equate learning speed with intelligence, but the fact that humans do not top the chart should not be one of them.’ Big tent accounts of cognition, by contrast, such as the one adopted here, are phylogenetically inclusive in that they focus on some widely and continuously distributed property that permits cross-species comparisons and generates evolutionary hypotheses. On the present account, cognition includes any information processing that occurs in the organism and enables it to interact with its environment. This encompasses not only centralized information processing that occurs in brains, but also information processing that takes place in peripheral nervous systems, perceptual modalities and non-neural cellular systems of representation.^1^

Such a phylogenetically inclusive account is controversial. Many cognition theorists have attempted to distinguish cognition from perception, for instance, by restricting cognition to certain types of information processing, formats of representation or degrees of encapsulation [14]. Nevertheless, an information-processing account is broad enough to capture many or all types of cognition, and can fit into an evolutionarily ecological framework that explains why cognition exists in the forms and taxonomic distributions that it does without ruling out the possibility that more complex forms of cognition may be found in organisms that are distantly related to humans. Such a big tent conception of cognition does not, however, provide us with the conceptual resources to distinguish intuitively simpler from intuitively more complex forms of cognition, because information processing is a continuous phenomenon that encompasses both higher-level and lower-level processes. What the big tent conception does provide is a phylogenetically inclusive theoretical foundation that stands the best chance of unifying and explaining the assorted mechanisms and processes that are plausibly characterized as cognitive and which enable organisms to interact successfully (and often flexibly) with their environments. ‘Phylogenetically inclusive’ here means more than the mere methodological injunction to keep an open mind with respect to in which branches of the tree of life particular cognitive mechanisms may be found. It means understanding cognition as ubiquitous in the living world, much like replication and metabolism—even if varieties of cognition, like forms of metabolism and replication, vary widely across the whole of life. This big tent account of cognition can serve as a unifying framework for explanations of all cognitive forms, from ‘simple’ to ‘complex’, and the behaviours to which they give rise—a framework that underpins the model of cognitive-behavioural evolution proposed in §4.

At present, it is unclear whether the types of cognition typically identified in the comparative cognition literature as comparably complex—and on the basis of this supposed complexity accorded a weightier burden of proof in hypothesis adjudication (§3)—have any common properties by virtue of which they can meaningfully be classified as ‘complex’. It is possible—though far from definitive—that the representational forms underwriting cognitive mechanisms intuitively considered complex differ from putatively simpler forms of information processing (such as perception) in terms of their degree of stimulus-independence, recombinability and/or encapsulation [15]. Such cognitive properties may be especially important in generating flexible behaviour, as more significant than the sheer amount of information an organism is capable of processing is *how* that information is packaged or encoded [16,17] and made accessible to other cognitive processes. Not only do some coding formats make it possible to process a greater total quantity of information, but they also allow for qualitatively different representational forms—such as planning, concept-formation, causal reasoning, intentionality, mindreading, metacognition and episodic memory—processes that may underwrite important types of behavioural flexibility observed in the field and elicited in the laboratory [6].

Much more could obviously be said about information processing accounts of cognitive mechanisms standardly identified by the comparative psychology community as complex. For present purposes, we remain agnostic as to whether any unifying theory of such mechanisms—and more particularly, of what makes them comparably *complex*—can be found. There are serious difficulties confronting attempts to conceptualize and operationalize biological complexity in general—difficulties that are only magnified in the cognitive realm. Yet despite these manifest difficulties with complexity talk, this paper retains the term because it does substantive theoretical and methodological work in comparative cognition research. As will be discussed in more detail shortly, it is precisely because the cognitive abilities mentioned above are typically regarded as relatively complex that they are treated as requiring higher burdens of proof than supposedly simpler hypotheses, particularly in the context of non-linguistic organisms (including developing humans). When this paper refers to ‘cognitive complexity’, therefore, it merely intends to pick out this intuitive usage. It remains neutral as to whether the cluster of cognitive abilities typically identified as complex (i) falls along a continuum of complexity or is structured by discrete, scalar jumps, (ii) is theoretically unifiable or represents a natural kind class [6,18], and (iii) is underpinned by a notion of complexity that is operationalizable in the cognitive case. Indeed, it is scepticism about the last point that will motivate our suggestion that the preference for simplicity be modified or abandoned (§3).

Unlike cognition, which has a wide-ranging and dedicated literature, far less has been written about the nature of behaviour, and even less about behavioural flexibility. One might initially question whether behaviour can fruitfully be described in a-cognitive terms. That is, even if one heeds our recommendation not to build complex cognitive mechanisms into the definition of behavioural flexibility, the problem remains that many behaviours are commonly delineated in reference to their underlying cognitive mechanisms. This is true not only for intentional actions in adult humans, but also for many non-human animal behaviours that are commonly described in loosely intentional terms. For example, when describing behaviour, we often say that an animal is ‘reaching for X’ rather than ‘extending a limb and touching X’. The prevalence of the former type of description of animal behaviour, which appears to incorporate particular cognitive mechanisms, is explicable in two ways—neither of which poses a problem for our view that complex cognition and behavioural flexibility should be definitionally distinct. First, the former behavioural description may simply serve as shorthand for the latter less economical one, in which case the former description could be cashed out in purely behavioural terms. Alternatively, the former behavioural description may very well incorporate particular cognitive mechanisms, but do so out of the general recognition among animal behaviour scientists that scientific objectivity no longer requires a behaviourist's exclusive commitment to observation-statements. What is crucial is not that behaviour be described in wholly behaviourist terms, but that the specific cognitive mechanisms under investigation are not incorporated into behavioural descriptions if the latter are to serve as evidence for those mechanisms.

What, then, do we mean by ‘behavioural flexibility’? For present purposes, behavioural flexibility shall be understood as a distinct type of behavioural plasticity, which in turn is a distinct type of phenotypic plasticity.^2^ Nearly all behaviours are minimally plastic in the sense that they are produced in response to particular stimuli, and these stimuli are only sometimes present in the environment. For instance, male fruit flies universally initiate a rigid sequence of courtship behaviours only when they detect pheromones emitted by a receptive female [20]. There is an important sense in which an organism that can perform a half-dozen behaviours, even if it does so rigidly in response to stimuli, is more plastic or versatile than an organism that performs fewer rigid behaviours. If we conceive of cognition in information processing terms, then we can already see the beginnings of a robust theoretical connection between cognition and behavioural plasticity. However, only a subset of behaviours are properly plastic—that is, governed by conditions of expression that allow the behaviour to be modified in response to environmental input, and only a subset of properly plastic behaviours are *flexible,* or modifiable throughout the lifetime of the organism based on experience.

In essence, then, behavioural flexibility is a special type of behavioural plasticity in which the rules governing behavioural expression, and thus the behaviours themselves, can be modified in accordance with environmental input throughout ontogeny [21].^3^ It is the alterability of the rules governing the expression of a behaviour, more so than the fact of environmental sensitivity *per se*, that allows organisms to respond in robustly flexible ways to the vagaries of their selective environment.^4^ This notion of behavioural flexibility is broader than those occasionally found in the literature (some of which are limited to, e.g. reversal learning),^5^ and includes behavioural repertoires that are often elicited in experiments to probe for complex cognitive abilities such as vocal learning and causal reasoning.

Not all experiments designed to probe for complex cognition in animals appear to elicit flexible behaviour in the sense defined above. For instance, mindreading and self-recognition experiments do not seem to test for learning abilities at all. Although some metacognition experiments do not test for behaviours that exhibit learning *in the course of the experiment*, they do test for capacities that are likely to have been learned through complex interactions with the social and physical world, and that derive from cognitive mechanisms causally implicated in flexible behaviours, such as perception-independent representations [24] and a sense of agency [25]. We may therefore regard the behaviours elicited in these experiments as flexible in an aetiological (diachronic) sense. Nevertheless, this paper does not intend to establish necessary and sufficient conditions for the presence of cognitive complexity, nor to provide an exhaustive account of complex cognition and its behavioural correlates; rather, its less ambitious aim is to provide a useful organizing schematic for investigating the link between cognition and flexible behaviour, which plays an important evidential role in animal cognition research.

The conceptual decoupling of behavioural flexibility and cognitive complexity allows the former to serve as meaningful evidence of the latter. Yet this leads directly to another obstacle that impedes an unproblematic inference from flexible behaviour to complex cognition: the problem of underdetermination in the field of animal cognition.

## 3. Underdetermination and the simplicity programme

Some philosophers of science have suggested that the underdetermination problem is primarily a philosophical concern, not one that is commonly borne out in the practice of science [26]. And yet, instances of apparent underdetermination are common in comparative cognition, with existing behavioural data appearing to be equally well confirmed by multiple incompatible cognitive explanations. For present purposes, it is not necessary to assert that this underdetermination is permanent or that it will remain unshaken by all possible sources of experimental evidence. It is enough to show that underdetermination exists relative to the actual evidence in hand, that it is pervasive in the science, that it is recurrent in the sense that equally well-confirmed alternatives are frequently available, and that this undermines our justification for believing that even our most well-confirmed hypotheses are true (see [27]).

Consider a well-known example from primatology: the case of chimpanzee mindreading. Chimpanzees are excellent predictors of conspecifics' behaviour, leading researchers to wonder whether chimpanzees understand that others have beliefs and desires (i.e. that they are capable of ‘mindreading’). Decades of studies, however, have resulted in a stalemate: while some interpret the experimental data as suggesting that chimpanzees are mindreaders, others note that the data are consistent with a putatively simpler ‘behaviour-reading’ explanation [28]. For example, according to Vonk & Povinelli [28], chimpanzees are merely responding to shallow behavioural cues without inferring mental states. Further, they reason that the experimental data support both mindreading and behaviour-reading hypotheses equally well, but that the behaviour-reading hypothesis is simpler, and therefore better. Philosophers reflecting on this stalemate have expressed scepticism that it can be resolved empirically (e.g. [29]).

Some comparative cognition theorists reject the assertion that both mindreading and behaviour-reading hypotheses are on equal epistemic footing in chimpanzee cognition research. For instance, Halina [30] argues that mindreading hypotheses are well tested, whereas behaviour-reading hypotheses are not actually probed by mindreading experiments. To represent genuine threats to the mindreading hypothesis, behaviour-reading hypotheses require independent sources of evidence; and yet they tend to be offered up ad hoc and then placed on equal or superior epistemic footing than the mindreading alternative. Halina's criticism is reminiscent of Gould & Lewontin's [31] classic critique of adaptationism, which targeted several argument strategies attributed to the so-called ‘adaptationist programme’. These include (inter alia) the tendency to readily replace one failed selectionist explanation with another, to have an unwarrantedly low evidential bar for accepting adaptationist explanations when compared with non-adaptationist ones, and failing to consider non-adaptationist alternatives at all.

Explanations that advert to lower-level cognitive mechanisms substitute for selectionist explanations in the critical analogy: just as ‘the range of adaptive stories is as wide as our minds are fertile, (and thus) new stories can always be postulated’ [31, p. 153], so too can lower-level cognitive explanations be invented for any behavioural datum. Likewise, just as consistency with natural selection is an inadequate basis for accepting adaptationist explanations, so too is consistency with lower-level cognitive explanations an insufficient basis for rejecting higher-cognitive ones. The key here, as in the evolutionary biological context, is to distinguish explanations that are on strong epistemic footing from among the sizable set of possible but less well-grounded explanations.

Nevertheless, there is one important element of Gould & Lewontin's critique of adaptationism that does not transfer to the present context: whereas the adaptationist programme was accused of ignoring non-selectionist explanations, lower-cognitive explanations are designed specifically to deflate (if not refute) higher cognitive ones by generating a situation of underdetermination. This problem of underdetermination is significantly exacerbated by a general preference for simpler explanations in comparative cognition, which poses a further methodological hurdle to establishing an evidential link between flexible behaviours and complex cognitive mechanisms. By a ‘preference for simpler explanations’, we mean the tendency in comparative cognition to reject higher-level cognitive explanations simply because lower-level ones cannot be ruled out [32]. In the context of the adaptationist programme discussed above, the analogous practice would be to reject any non-selectionist explanation no matter how well supported unless and until all plausible selectionist explanations have been excluded. In the ‘simplicity programme’ that is widely (though not universally) embraced in comparative cognition, putatively simpler hypotheses are treated as theoretical defaults that experiments must be able to exclude before more complex cognitive hypotheses can be accepted. In other words, explanations positing putatively simpler cognitive mechanisms should, all else being equal, be preferred over explanations that posit putatively more complex ones—an idea that is embraced in both comparative cognition [1,33] and in the psychological sciences more broadly [34]. This *a priori* preference for simplicity ‘resolves’ the problem of underdetermination by offering a clear strategy for choosing among empirically adequate hypotheses—one that places the burden of proof on the supposedly more complex hypothesis. In practice, however, this burden of proof is impossible to meet, as deflationary cognitive hypotheses—like their selectionist analogues—can rarely be ruled out definitively. Thus, the simplicity programme appears to require an unreasonably high standard of evidence for establishing complex cognitive explanations, and an unwarrantedly low standard of evidence for accepting putatively simpler ones.

What justifies the simplicity programme in comparative cognition? Typically, the preference for simpler explanations is justified by appealing to what is known as ‘Morgan's Canon’—a brief passage in a founding text of comparative psychology by C. Lloyd Morgan, which states:

In no case may we interpret an action as the outcome of the exercise of a higher psychical faculty, if it can be interpreted as the outcome of the exercise of one which stands lower in the psychological scale (1894: 53).

This textbook passage is frequently, though incorrectly, interpreted as a version of Occam's Razor, holding that one ought to prefer the simplest explanation consistent with the observed behaviour, barring compelling evidence to the contrary.^6^ By advising experimenters to select the simplest hypothesis as the ‘null’, the simplicity preference in effect elides the problem of underdetermination by shifting the burden of proof onto explanations that postulate more complex cognitive mechanisms.

It is far from clear that this is the right way of resolving the underdetermination problem in comparative cognition, however, given that the simplicity preference—and thus the simplicity programme—appears to rest on shaky conceptual, theoretical and empirical grounds [38,40–46]. Conceptually, there is no such thing as simplicity *simpliciter*. Rather, different scientific contexts call for different approaches to simplification and, consequently, different justifications of those approaches. Moreover, simplicity vis-à-vis explanation is very different from simplicity vis-à-vis the entities referred to in those explanations. The fact that a hypothesis is simple (on some operational semantic metric) does not entail that the entities that the hypothesis describes are also simple (on some operational ontological metric). The simplicity of semantic structures (as measured, e.g. by description length or maximum compressibility as described in Kolmagorov complexity theory) need not entail simplicity in the physical structures, causes or mechanisms postulated by those explanations (as measured, e.g. by the number of entities or entity types they feature). Because there appear to be numerous, mutually irreducible ways to simplify the world and our descriptions of it, we cannot expect all forms of simplification to yield equally desirable results from a methodological standpoint [46]. Thus, any broad-based preference for simplicity is likely to be too coarse-grained to be of scientific use.

Theoretically, the operative assumption that biological outcomes will tend to have simple rather than complex causes seems particularly ill-suited for the biological world. If natural selection is more like a historically constrained Rube Goldberg-style tinkerer, rather than an elegant optimizer [47], then we should expect functional complexity to be the norm and simplicity the exception at all levels of the biological hierarchy. Models of functional gene regulatory networks, for example, are typically daunting in their baroque mechanistic complexity. In such cases, causal simplicity is, quite rightly, typically not a key factor in model adjudication; in fact, the elegance of biological models will often undermine their real-world applicability [48]. Likewise, in studies of large-scale evolution, ‘stochastic’ models that postulate numerous complexly configured causes have for decades served as the null hypothesis against which so-called ‘deterministic’ models—those that postulate a single cause or a few major causes—are tested [49]. Although optimality models are commonly deployed in evolutionary biology, they typically serve as idealizations that allow us to measure the influence of non-selective forces in evolution [50], rather than arising from a broad ontological commitment to functional streamlining in nature. As animal cognition is an evolutionary outcome, it is incumbent on the defender of the simplicity programme to provide some empirical justification as to why we should treat cognitive systems differently from other biological systems in this respect.^7^

How might such an empirical case proceed? One potential empirical justification for the simplicity preference might appeal to the relative evolvability of simpler versus more complex cognitive solutions. One form of this argument, which has rarely been made explicit, is what might be called the ‘metabolic argument’. This holds that natural selection will, ceteris paribus, tend to favour the most metabolically frugal biological structures among those structures that can realize the same (or a sufficiently similar) function; further, because complex cognitive mechanisms are more metabolically demanding than less complex ones (because they necessitate more metabolically expensive neuroanatomical structures), we should err on the side of explanations that advert to simpler cognitive mechanisms in cases of underdetermination. Thus, the metabolic argument moves from an ontological claim that natural selection will tend to favour metabolically frugal structures among functionally equivalent (or sufficiently similar) alternatives, to the methodological claim that we should prefer a scientific methodology that biases against findings of cognitive complexity in circumstances of apparent underdetermination—which, in effect, is what the simplicity programme in comparative cognition is designed to do.

The metabolic argument demonstrates one way that evolutionary theory could potentially inform methods in comparative cognition. The argument is problematic, however, because it relies on claims about evolution in general—and cognitive evolution in particular—that are either unwarranted or underdetermined. At the most general biological level, it is clear that fitness trade-offs associated with the evolution of more energetic structures often favour metabolic increases. Indeed, the history of life on Earth is characterized by a robust trend toward increasingly energetic metabolisms, structures and lifeways [52]. More to the point, we know that metabolically costly brains have evolved independently in a wide range of taxa and that these metabolically costly structures have been retained in many of the taxa in which they evolved; further, it is generally presumed that these metabolically demanding neural structures subserve cognitive functions. Thus, the selective trade-off between a metabolically demanding substrate and the perceptual and cognitive gains it affords is often a favourable one. It follows that simpler cognitive solutions in many cases are not forthcoming because either (i) they are not more metabolically efficient than complex cognitive ones or (ii) they are more metabolically efficient but are not functionally equivalent (or sufficiently similar) alternatives to cognitively complex solutions. We suspect that both of these factors play a role in the evolution of complex cognitive mechanisms.

First, it is far from clear that increases in cognitive function require increases in brain tissue and, correspondingly, increases in metabolic expenditure. Higher-cognitive solutions may be achieved simply by repurposing relevant areas of the brain for novel tasks, leaving the total neural metabolic budget unchanged. Furthermore, some apparently simple cognitive tasks, such as association, may require significant computational and neurological power [53], while some increases in informational capacity (especially qualitative ones) may actually *reduce per capita* metabolic cost. Second, even if cognitively simpler mechanisms were on average less metabolically demanding than more complex ones, there is no reason to suppose that simpler solutions will be functionally equivalent to more complex ones—and if they are not functionally equivalent, then there is no basis for claiming that selection would prefer simpler over complex cognitive mechanisms in any given case. Although we can conceive of plausible lower-cognitive explanations for many observed instances of behavioural flexibility, this does not mean that the competing complex and simple cognitive mechanisms that could account for these instances of behavioural flexibility are themselves functionally equivalent. This is because each competing cognitive mechanism will be associated with a range of behaviours that impact on fitness, and these fitness impact ranges may not be equivalent. In other words, a behaviour witnessed in an isolated experimental setting may be part of a wide repertoire of behaviours underwritten by a complex cognitive mechanism, and taken as a whole this behavioural output range may have a higher relative fitness value than that produced by a comparably simpler cognitive mechanism. Therefore, the fact that a simpler cognitive mechanism can explain an isolated case of behavioural flexibility as competently as a more complex cognitive mechanism does not mean that, from an evolutionary standpoint, the two solutions are functionally equivalent—and thus there is no sound evolutionary basis for preferring the simpler one. In sum, there are simply too many unanswered questions and too many unfounded evolutionary assumptions here for a metabolic rationale to support a robust, context-insensitive, course-grained methodological bias against explanations that advert to more complex cognitive mechanisms.

However, the metabolic argument is not the only evolutionary argument that could support the simplicity programme. Another, which might be called ‘the fast and the frugal’ argument, focuses on the comparative performance advantages of simple over more complex forms of cognition irrespective of their metabolic requirements. For example, simple strategies have been shown to outperform more complex cognitive strategies in decision-making and problem-solving contexts in terms of both speed and accuracy [54]. These fast and frugal strategies work when the environment is structured such that it can be easily exploited for the rapid retrieval of ecologically relevant information. This is the case, for example, when the environment contains stable and relatively simple patterns that organisms are capable of detecting and which support simple heuristics; it may also be the case in unpredictable, noisy and/or complexly configured environments (such as stock markets) in which tracking a small set of salient cues (such as the behaviour of well-known stocks) may be a superior strategy to complex optimizing models that search for patterns among all available information [55]. If fast and frugal strategies are adaptive, then at least when environments are appropriately structured, we should expect organisms to evolve such cognitive shortcuts, rather than investing in slower and perhaps less reliable general-purpose mechanisms. Taken to its methodological conclusion, the fast and the frugal argument asserts that scientists ought to privilege explanations that advert to simple heuristics (such as feature extraction) over those that postulate more complex cognitive mechanisms whenever experimental evidence supports both explanations equally well. This, in turn, would allow the simplicity preference to act as a rational tie-breaker in cases of underdetermination, and provide a strong corrective even in cases where the evidence in favour of a more complex cognitive mechanism begins to mount.

As with the metabolic argument, the fast and the frugal argument cannot justify a blanket preference for simpler explanations. First, the argument only holds for lineages whose environment is appropriately informationally structured; at present, however, we do not know how pervasive such informational ecologies are—or how applicable some of the human-focused cases, such as the stock market, are to the ecologies of other organisms. Thus, our current state of knowledge does not support a sweeping preference for simpler cognitive explanations by way of the fast and the frugal rationale. Second, even if an animal tends to make use of simple heuristics in some cases, it may still be capable of switching to a more cumbersome cognitive strategy that it keeps in reserve for situations in which simple heuristics fail or are silent. For instance, while a student may not typically reason using predicate logic, she may switch to logical reasoning when faced with writing a philosophy paper. Similarly, a rat may toggle between simple rules and metacognitive strategies, or a bee may toggle between feature extraction and template matching. In sum, while it may be reasonable to say that ceteris paribus, natural selection will tend to favour simpler cognitive mechanisms, so many evolutionary, ecological and developmental assumptions are packed into the ‘ceteris paribus’ clause that the statement is, if not vacuous, then far too course-grained to license an evidential burden of proof in any given case.

Not all empirical justifications of the simplicity preference will appeal to evolutionary considerations, however. It might be argued, for example, that simpler explanations (e.g. behaviour reading) are on stronger epistemic grounds than their more complex alternatives (e.g. mindreading) because we already know that the animals in question have the simpler ability but we are not certain that they have the more complex one. This line of reasoning is, in fact, circular: it draws upon an evidence base that was itself established by methods that may have privileged the simpler hypothesis [38]. In other words, because many experiments have followed the simplicity programme, much of the evidence for cognitive abilities may be skewed toward simpler explanations, and this possibility should weaken our confidence in the simpler alternative. In any case, this rationale turns not on comparative simplicity, but on pre-existing evidence that some explanatorily adequate capacity is present in a given lineage while the existence of another explanatorily adequate capacity is less certain. Thus, on this empirical rationale, simplicity does none of the adjudicatory work.

For all these reasons, a strong *a priori* simplicity preference is not a preferable solution to the underdetermination problem. As Elliott Sober [56] and Simon Fitzpatrick [42,57] have argued, the only legitimate tie-breaker when experimental data cannot adjudicate among competing hypotheses is more evidence. The burden of proof should favour not the simplest hypothesis, but the best evidenced hypothesis. This can be accomplished by modifying the null hypothesis so as to build into it probability-conferring evidence [58]. Such evidence would include not only prior experimental evidence but also evolutionary, phylogenetic, ecological, neurobiological, developmental and behavioural data about the species in question. Thus, a more promising solution to the underdetermination problem in comparative cognition—and the most plausible way of establishing an evidential link between flexible behaviour and complex cognition—involves looking to evidence outside of the experimental context. Put differently, it entails shifting our focus from the ‘context of discovery’, in which external evidence has no power to adjudicate among hypotheses, to the ‘context of justification’, in which all relevant bits of evidence are factored into the null hypothesis (or prior probability) that bears on a given conclusion. In essence, comparative cognition would benefit by diversifying its methodology, drawing upon disparate streams of evidence, and correcting for *a priori* biases toward simplicity that have biased experimental work against findings of complex cognition and thus shaped all-things-considered judgements about the nature of cognition and its phylogenetic distribution. What would this extra-experimental evidence base look like, in what ways can it inform experimental practice, and how might it substantiate a link between behavioural flexibility and complex cognition? Sketching a preliminary answer to this three-part question is the task of the next section.

## 4. Beyond the experiment: an adaptive triadic model of cognitive-behavioural evolution

Our aim in this section is to outline a model that demonstrates how evidence in evolutionary biology can support the inference from behavioural flexibility to cognitive complexity, and thereby bear on theory adjudication in comparative cognition science. A broad-scale picture of macroevolution suggests that the emergence of increasingly flexible behaviour in animals is closely tied to the evolution of complex information processing mechanisms realized by nervous systems. On the most plausible reconstructions of metazoan evolution, cognitive complexity has arisen independently in groups as developmentally diverse as vertebrates, molluscs and arthropods [59]—clades that boast complex image-forming sensory capabilities and central nervous system functions that support rich sensorimotor information flows, which in turn underwrite the most rapid, sophisticated and flexible behavioural repertoires in the living world.

The theoretical foundations of this non-accidental regularity can be found in Godfrey-Smith's [21] ‘environmental complexity thesis’, which holds that the evolutionary function of cognition is to enable organisms to interact in fitness-enhancing ways with a heterogeneous environment (see also [60]).^8^ Cognitive processes do this, on Godfrey-Smith's account, by exploiting ecologically relevant information. Cognition only gets its purchase, therefore, when there is both heterogeneity in the environment and reliable cues of ecologically relevant variations. Despite its theoretical plausibility, the environmental complexity thesis is difficult to test, in part because the concept of heterogeneity is difficult to define and operationalize. As Godfrey-Smith concedes, it is unlikely that we will be able to articulate a general definition of environmental heterogeneity that is both testable and permits cross-taxa comparisons. For example, we may be able to compare the relative environmental heterogeneity for honeybees to that of mosquitos, because both are flying insects, even if we cannot do so between more distantly related species such as monitor lizards and hummingbirds. In this respect, the environmental complexity thesis is akin to the principle of natural selection: an organizing schematic that helps to unify (and thereby explain) a wide range of cases, but one that is not testable until lineage-specific ecological, developmental and evolutionary parameters are filled in [8]. For present purposes, we will rely on the following provisional understanding of *environmental heterogeneity*:

Environment A of evolving lineage X is more heterogeneous than environment B of evolving lineage Y only if A contains more fitness-relevant informational cues in relation to X (given the developmental parameters of X) than B does in relation to Y (given the developmental parameters of Y).

Here, ‘fitness-relevant informational cues’ refers to informational cues that would, if detected and acted upon, have some net statistical effect on organismic fitness. For example, an animal's social environment is more heterogeneous the more types of calls, postures and conspecific interactions it needs to keep track of. Fitness-relevant informational cues are indexed to the evolutionary developmental parameters of particular lineages to acknowledge a degree of organism–environment codetermination, wherein the organismic features of lineages actively shape their selective environments [8,62].

On the adaptive model of cognitive-behavioural evolution that we propose, complex cognitive mechanisms will fail to evolve, or, if they already exist, begin to degrade due to the relaxation of stabilizing selection, in environments where either: (i) there are few fitness-relevant informational cues, (ii) there are many fitness-relevant informational cues, but cheaper solutions (such as camouflage or simple cognitive heuristics) are readily accessible, or (iii) there are many fitness-relevant informational cues but some evolvability constraint (resulting, e.g. from the lack of a nervous or motor system, a prohibitive trade-off, or a complexly configured/dynamic informational environment that makes it difficult to extract relevant cues) prevents the requisite phenotypic variations from arising. Conversely, we may expect that, ceteris paribus, behavioural flexibility will arise when animal lifeways incentivize the detection and processing of a wide range of informational cues whose natures and sources vary substantially over space and time. For instance, if a generalist predator is confronted with prey types that vary widely over time and geographical range, they will, barring constraints, tend to develop flexible strategies of predation, which will sometimes be underwritten by more complex cognitive mechanisms. By contrast, lineages that utilize only one or a few stable environmental resources—such as many grazing herbivores, be they arthropods or vertebrates—can be expected to process comparably less information about their environment, and as a result will tend to exhibit more rigid behaviours and comparably simple neural machinery (e.g. grasshoppers, sauropods, bovids, koalas, etc.).

On Godfrey-Smith's view, the environmental complexity thesis serves as a model that can be applied on a case-by-case basis and, if it provides a successful account of the evolution of cognition in numerous instances, then it may be generalized to a still broader range of cases. We propose something similar in working toward an account of the wider evidential context in which competing hypotheses in comparative cognition may be adjudicated. According to what we will call the ‘adaptive triadic model’ of cognitive-behavioural evolution (henceforth ‘ATM’), three elements—behavioural flexibility, environmental heterogeneity and the neuroanatomical structures associated with increasingly sophisticated information processing—serve as conceptually independent and mutually reinforcing sources of evidence that indicate the presence of cognitive mechanisms generally regarded as complex. This information can then be incorporated into the null hypothesis or prior probabilities against which experimental data are weighed. When taken in isolation, observations of behavioural flexibility have limited evidentiary power; once other components of the ATM are included, however, the weight of behavioural flexibility as a source of evidence for cognitive complexity increases substantially. The effect of incorporating these other sources of evidence is not to lighten the evidential load that behavioural flexibility is expected to bear, but rather to strengthen it. In other words, the ability of behavioural flexibility to serve as strong evidence of complex cognition is context-sensitive, and this context is provided by other components of the ATM. Behavioural flexibility continues to play a special evidential role, however, as inferences of cognitive complexity will generally be untenable if they are not supported by any behavioural observations whatsoever (though neurological and ecological data could provide grounds for further behavioural investigation).

While elements of the ATM serve as conceptually independent sources of evidence for cognitive complexity, they are not *causally* independent of one another—indeed, it is precisely in virtue of their evolutionary casual interdependence that they have predictive power. More precisely, what gives the ATM traction is the idea that the convergent evolution of trait–environment pairings in phylogenetically disparate groups of organisms can constitute ‘natural experiments’ [63] that support the existence of non-accidental (or law-like) regularities, which in turn can be used to inform work in comparative cognition. Convergent evolution is considered among the strongest evidence for adaptive hypotheses [64]: for instance, the fact that both ichthyosaurs (Mesozoic marine reptiles) and dolphins independently evolved dorsal and tail fins in a fully aquatic environment strongly suggests that these structures are adaptive and that they have similar evolutionary functions in each case. Currie [65,66] provides a helpful schematic for such an inference, according to which a known pattern of convergence enables us to project a certain trait combination observed in a ‘model’ lineage onto a ‘target’ lineage that is known to exhibit some (but not all) of the traits in the model cluster. What allows for this projectibility, Currie suggests, depends on the relation between the model and target lineages. In the case of homology (ancestral similarity) relations, trait inferences are justified by the reliable inheritance of developmentally interconnected characters: if certain complex cognitive mechanisms are shown to be present in one lineage, then they may be inferred to exist in a closely related lineage, unless additional evidence suggests otherwise. In the case of convergence, trait inferences are justified by a biological regularity caused by a shared selection regime or other common forces that are ‘external’ to the lineages in question.

Given this inferential schematic, convergent regularities enable us to infer certain traits on the basis of particular selective environments, certain selective environments from the presence of particular traits, and certain traits from the existence of other traits in a non-accidental trait cluster given a particular selective environment. For instance, say we know that dorsal fins and tail fins form an iterated (convergent) trait cluster in connection with fully aquatic vertebrate lifestyles, such that we can expect to find dorsal fins in a new species of marine vertebrate if we know that it has tail fins. The ATM is premised on a similarly robust convergent regularity, in this case one that includes broadly defined behavioural traits, their proximate cognitive causes, the neural signature of these proximate causes described at an appropriate grain of resolution,^9^ and a heterogeneous selective environment that poses design problems to which behavioural flexibility is a solution.

The fact that elements of the ATM are historically causally interdependent does not mean that they are conceptually intertwined in a way that undermines their ability to support inferences concerning synchronic cognitive capacities. For instance, how heterogeneous an environment is will be determined in part by the sensory modalities, cognitive capacities and neurological structures of the lineage in question. As lineages evolve in cognitive (including perceptual) sophistication, so too does the extent and type of environmental heterogeneity they encounter. Acknowledging that the developmental parameters of lineages shape their selective environments does not pose conceptual or methodological problems for assessing environmental heterogeneity [8] or for allowing neurological and ecological data to serve as evidence of cognitive complexity by way of the ATM cluster. In fact, elements of the ATM are likely to evolve in feedback with one another: the emergence of novel cognitive abilities may open up ecological opportunities that increase the total number of fitness-relevant informational cues, which in turn drive the evolution of more sophisticated neural structures and cognitive mechanisms in ratchet-like fashion (see [43]).

The more the ATM regularity holds across distant animal groups with disparate developmental plans, the less likely it is to be the product of chance or quirky features of particular groups, and the greater the likelihood that the traits cluster together non-accidentally due to forces or constraints that are external to the converging lineages. Unlike projections based on homology relations, which are generally limited to closely related taxa, the ATM permits inferences across large expanses of the tree of life. This is particularly important in the case of comparative cognition, because centralized or otherwise massively augmented information processing centres have arisen independently numerous times in protostomes and deuterostomes, and thus any regularities that subsume these cases cannot be grounded in (or *solely* in) homology. Although these iterated outcomes are produced in part by conserved developmental resources, such as deep homologues, cell types and/or patterning mechanisms that were likely present in the ancestor of *Bilateria*, the neural proliferation and much of the architectural organization that characterizes these events is convergent [69].

If the ATM is correct, then we should find evidence of the hypothesized trait–environment cluster across phylogenetically distant linages. Indeed, there is a growing body of evidence linking the enlargement and/or increase in neuron density of brain regions that are causally associated with informational integration in mammals (the cortex including the neocortex and striatum), birds (the telencephalon including the neostriatum and hyperstriatum ventrale), octopuses (the vertical lobe) and insects (the mushroom bodies) to problem-solving abilities that evolved in the context of heterogeneous selective environments (table 1) [4,87,88]. Further phylogenetically broad evidence, gathered in table 1, supports these findings.

**Table 1.** Support for the adaptive triadic model from examples where similar data were collected on closely related species. Species that demonstrate better problem-solving abilities had more opportunities to learn from previous experience (*flexibility*; italics) through a more ***heterogeneous environment*** (bold italics), and show enlargements in causally related **brain structures** (bold).

taxa	species	description of species comparison
mammal	*Pan troglodytes, Papio anubis*	chimpanzees are better at solving *spatial and tool use* tasks, have a larger **neocortex** relative to their total brain size, and their ***arboreal*** lifestyle results in a more heterogeneous environment than ground-dwelling olive baboons [70–72]
mammal	*Crocuta crocuta, Parahyaena brunnea, Hyaena hyaena, Proteles cristata*	spotted hyenas can solve a *puzzle box* that striped hyenas cannot, and they have the most ***complex social system***, ***hunt the largest prey***, and have the largest **anterior cerebrum** volume (part of the frontal cortex) [73,74]
bird	*Molothrus bonariensis, M. rufoaxillaris, M. badius*	brood parasitic screaming and shiny cowbirds have larger **hippocampuses** than non-brood parasitic bay-winged cowbirds, probably because brood parasites need better *spatial memory* to remember ***where host nests are and when they might be ready for parasitic eggs to be laid***. Female shiny cowbirds had larger **hippocampuses** than male shiny cowbirds, probably because only the female ***searches for nests*** in this species. There were no sex differences in hippocampuses or search behaviour in screaming cowbirds [75]
bird	*Ailuroedus crassirostris, Scenopoeetes dentirostris, Prionodura newtonia, Ptilonorhynchus violaceus, Chlamydera nuchalis*	bower building species had larger **telencephalons** (minus the hippocampus) than the non-bower building catbird. Among bower building species, *bower complexity* increased with **cerebellum** size (responsible for motor learning [76]). Catbirds feed their offspring fruit (mostly figs) rather than insects as the other species do, and the ***fruits*** the bower building species rely on are patchily distributed in the non-breeding season [77]
fish	*Bathygobius cocosensis, B. krefftii, Favonigobius lentiginosus, Istigobius hoesei*	***rock dwelling*** gobies (Cocos frillgoby and Krefft's goby) learned a *spatial* task faster, made fewer errors [78], and have larger **telencephalons** [79] than sand-dwelling gobies (eastern long-finned goby and Hoese's sandgoby)
fish	*Labroides dimidiatus*, compared with 24 other species in the same order	the cleaner wrasse fish engages in complex *social interactions* driven by ***repeat interactions*** with the same clients (fish that the cleaner wrasse clean), and has one of the largest **diencephalons** (one of the brain regions responsible for social decision-making) compared with 24 other species in the order Perciformes [80,81])
insect	*Apis melifera, Bombus impatiens*	bumblebees originated in the temperate latitudes where ***flowers are more patchily dispersed*** than in the tropics where honeybees originated. Bumblebees *socially learn* about nectar robbing and can adaptively reverse a previously learned preference, while honeybees do neither [82]. Bumblebees have larger relative **mushroom bodies** than honeybees [83]
cephalopod	*Nautilus pompilius,* octopus, cuttlefish	octopus and cuttlefish have excellent *spatial navigation* abilities, short and long-term *memory* in associative learning tasks, their brains have **vertical lobes** (where learning and memory are processed), and they are highly mobile and pursue ***mobile, patchily distributed prey*** when compared with the nautilus which has poor long-term memory, lacks vertical lobes, and forages by scavenging [84–86]

An additional source of evidence for the ATM comes from a similar regularity in ontogeny: cases where complex cognition, flexible behaviour and neuroanatomical complexification correlate with fluctuations in environmental heterogeneity within the lifetime of an organism. For instance, London taxi drivers who must navigate a spatially heterogeneous environment enjoy better-than-average spatial navigation abilities, and have been found to have a correspondingly larger posterior hippocampus relative to average humans [89] (for other examples see [90–102]). Although this pattern is ontogenetic, it supports the ATM for two reasons. First, it shows that heterogeneous environments call for more flexible behaviours and that these, in turn, require additional neuronal growth. If this link is present in ontogeny, we can expect natural selection to also exploit the link over evolutionary time. Second, the mechanisms through which the ATM is established at evolutionary scales could exploit some of these ontogenetic effects. This could occur, for example, through a process of ‘genetic assimilation’ whereby plastic phenotypic variation becomes environmentally canalized so that it comes to be produced without the environmental stimulus.

If the ATM is borne out empirically, then behavioural flexibility could serve as evidence of cognitive complexity for lineages that evolved in heterogeneous selective environments and exhibit relevant neural structural variations. The ATM could also justify treating a cognitively complex hypothesis as the null against which putatively simpler cognitive explanations bear the burden of proof. By contrast, where critical traits in the cluster are lacking—such as the relevant neuroanatomical correlates, environmental heterogeneity or behavioural flexibility—inferences to or methodological biases in favour of cognitive complexity will be on shakier grounds. In conjunction with inferentially rich homology data [103], the ATM offers a promising source of evidence beyond the laboratory that can inform methods and theory adjudication in comparative cognition research.

There is a potentially important disanalogy, however, between the evidential schematic presented by Currie [66] and the kind of inference-making contemplated here. In Currie's model of inference, *observed* phenotypic traits of a model lineage are projected onto a target lineage on the basis of their homologous or convergent relations to the target. By contrast, the present case involves traits—cognitive mechanisms—that have never been directly observed in *any* animal lineage. Thus, we are projecting an *unobservable* trait inferred in a model lineage onto a target lineage. This is appropriate, however, because the projectibility of a given trait in any trait cluster–environment regularity hinges not on direct observability of the trait, but rather on our epistemic warrant for believing that the trait exists in model lineages. The fact that cognitive sophistication is not directly observable need not be a problem for our model, so long as cognitive complexity has been reliably inferred in a sufficiently large number of cases.

Another concern may be that the ATM is viciously circular insofar as each source of evidence lends independent weight to the others. How can trait X be evidence of trait Y, Y be evidence of trait Z, and Z be evidence of X, without circularity? The answer is simple: what grounds inferences such as ‘If X, then (probably) Y’ where X and Y are non-accidentally clustering traits is that they both stem from a common cause. In some cases, this common cause is inheritance from a common ancestor. In the case of the ATM, the common cause is selection in a broadly common ecological regime, along with structural and physiological constraints on the ways that complex cognitive solutions can be realized [69,104]. For the same reason, the inference ‘If X, Y and Z then (probably) environment E’ may be justified where E is a common cause of X, Y and Z.

There are two additional circularity worries that are not dispatched by the above common cause argument. The first is that the ATM may be used to generate evidence in its own favour, leading to a circularity problem analogous to that of the simplicity programme (§3), wherein the results of a biased method are used to justify the method's bias. The worry is that if future research employs the ATM in order to identify complex cognition, then it will bias findings in favour of cognitive complexity, which could then illicitly be used to bolster the ATM regularity. However, the ATM does not bias research in favour of complex cognition attributions; to the contrary, it can serve as evidence both *for* and *against* findings of complex cognitive abilities on a case by case basis, depending on which features of the cluster are present (or absent). For instance, where flexible behaviour is found in the absence of a heterogeneous environment and relevant neural underpinnings, the ATM cautions against hasty attributions of complex cognition. The model therefore does not import a context-insensitive bias in favour of cognitive complexity in the way that the simplicity programme does for simpler cognitive mechanisms. Another circularity worry is that findings of cognitive complexity involve inferences to unobservable entities, and these unobservables are not directly supported by the ATM, as the latter only licenses inferences from one observable feature of the cluster to another. This concern can be put to rest as well by noting that the evidence drawn upon in support of the ATM, which is enough to get the model off the ground, is derived not from ATM-licensed inferences, but from experimental findings that were arrived at in an epistemic environment that was, methodologically speaking, quite hostile to findings of complex cognition. Thus, support for the ATM does not come from the ATM itself, and hence the model is not problematically circular.

One might further query whether an evolutionary account like the ATM, which identifies an *aetiological* regularity, sits at the wrong level of explanation when it comes to identifying the *proximate* mechanisms at work in animal cognition. It is true that if we had a full understanding of the synchronic causal structure of cognition and could reliably infer this structure from neural anatomy and/or behaviour, we would have no need to draw upon diachronic information provided by evolutionary regularities like the ATM. But such an understanding, if attainable, lies well beyond the horizon of our present knowledge. Disciplines working under conditions of substantially incomplete information—which is the case with comparative cognition/neuroscience as much as it is with historical sciences like palaeontology—should engage in what Currie [105] calls ‘methodological omnivory’. This entails making use of all epistemic resources at our disposal to develop theories about phenomena that are not directly observable. In the case of comparative cognition, methodological omnivory involves looking beyond the epistemic confines of behavioural experiment, and drawing on evolutionary concepts and methods to make inferences, inform hypothesis testing, adjudicate theories and delineate the functions of brain structures. This is precisely what the ATM is designed to do.

While the preliminary evidence in table 1 supports the ATM, there are also cases that appear to contradict it. For instance, the giant panda enjoys a monotonous foraging ecology that requires relatively little information processing, suggesting that panda brains should be proportionally small and simple; yet, the panda boasts a larger than predicted brain size for its body size [106]. Such cases may present as counterexamples to the ATM until one realizes that (i) the ATM describes only statistical, not absolute, regularities, (ii) some putative counterexamples, on closer inspection, turn out to be consistent with the ATM, and (iii) the ATM obtains at courser grains of phylogenetic and neuroanatomical resolution and may break down at finer grains because it becomes swamped by historical factors (such as phylogenetic inertia, as discussed below).

The first point is that, as with other postulated mechanisms and regularities in evolutionary biology, the ATM is a statistical thesis rather than an invariant, exceptionless law—and thus it is not refuted by a small number of counterexamples. The key question is not one of existence but of relative significance [47]. The second point is that some glaring counterexamples to the ATM turn out to support it. Take, for instance, the social brain hypothesis [107], which predicts that heterogeneous social structures tend to lead to evolutionary increases in brain size that underwrite behavioural flexibility, which enables organisms to navigate their complex, variable social landscapes. Although there is empirical support for this hypothesis,^10^ there are also apparent counter-examples. Consider the surprising finding that highly social ants and bees are no more encephalized than their solitary wasp ancestors [108]. We can infer from phylogenetic and fossil data that the markedly encephalized mushroom bodies of hymenoptera evolved many millions of years prior to the emergence of eusociality in these groups [109]; similar patterns are seen in the evolution of eusocial termites, which exhibit significant reductions in brain complexity as compared to their asocial, generalist-foraging cockroach ancestors [110]. These findings seem to cut against the social brain hypothesis and by implication the ATM, until one realizes two things.

First, encephalized mushroom bodies in hymenopterans arose in central-place foraging parasitoid wasps whose heterogeneous ecology required greatly expanded spatial memory and learning capacities—enhanced information-processing capacities that may have been subsequently coopted for the complexities of eusocial living and, as a result, did not require additional encephalization. Second, the evolution of highly specialized eusocial insect colonies reflects the emergence of a new evolutionary individual, which in some cases will entail reduced ecological heterogeneity for members of specialized castes and, thus, not result in an increase in neural architecture [111]. Indeed, the evolution of individuality is characterized by the specialization of parts within the individual (via, e.g. epigenetic modification), which results in a reduction of functional complexity within those parts because many functions can now be offloaded onto the larger collective [112]. So what looks initially like an exception to the ATM can in fact be accommodated by the model.

The third point is that other apparent exceptions to the ATM can be explained by ‘phyletic inertia’, or constraints on future evolutionary directions imposed by earlier body plan adaptations [31]. For example, the panda's anomalously large brain size (mentioned above) is probably due to the evolutionary recentness with which the panda—a clade within Ursidae—adopted its derived, ecologically homogeneous lifestyle. We might expect a similar anomalous pattern for other secondarily herbivorous clades, such as therizinosaur theropod dinosaurs and the jumping spider *Bagheera kiplingi*. This example illustrates another way in which diachronic evolutionary theory can help inform synchronic understandings of animal cognition: the panda's homogeneous environment, coupled with the principle of phyletic inertia, allows us to predict that the giant panda may not be utilizing its large brain in the same way its ancestors did, even if some neural (and perhaps cognitive) vestiges of that evolutionary history remain due to homology. Thus, neurobiological evidence alone, at least in anything approaching its current grain of resolution, does not provide high powered predictions of the extant cognitive abilities of clades—we need to supplement these data with aetiological and ecological information.

Another concern about the model proposed here is that it is too abstract, or the regularity it describes too course-grained, to inform any specific cases in comparative cognition. It is true that the ATM does not, on its own accord, provide evidence for the presence of *particular* cognitive mechanisms. Nor does it predict precisely which sorts of flexible behaviours were selected for in any given case. Although the selective pressures underlying the convergent evolution of neural circuits that subserve specialized sensory functions may readily be identified, it is difficult to determine the selective causes of convergent elaborations of neuroanatomy that could subserve functions for complex cognition. However, this is not the evidential use to which the model is intended to be put. Rather, the ATM merely licenses the inference that *some* complex cognitive mechanisms have evolved; this then supports, over rival simpler cognitive explanations, the complex cognitive mechanism that best explains the behavioural data in any given case, which then informs the choice of a null hypothesis.

Finally, one might worry that too little is known about comparative neuroanatomy to make any bold claims about the physical substrates of cognitive complexity. We agree that one must be cautious when drawing on apparent neuroanatomical analogies, as data are indeed sparse for many species and the means of mapping cognition onto brains is notoriously difficult.^11^ However, the ATM will hopefully stimulate further research and is open to adjustments in its neuroanatomical parameters as novel data are incorporated and concepts and methods are refined. Where enough is known about the organism in question, the ATM may aid experimental work in comparative cognition by informing the choice of an appropriate null hypothesis, as illustrated in box 1.

Box 1. **Case example. Hunting behaviour of the *Portia* jumping spider.**

*Goal*: Suppose that we wish to understand how *Portia fimbriata* succeeds in hunting a larger web-building spider, *Zosis genicularis*.

*Selecting a null hypothesis*: We must first select a ‘contextual null’ hypothesis—i.e. one that draws on background theoretical and empirical knowledge [58,115]. This requires looking beyond controlled experimental data to consider what the ATM predicts in the case of *Portia*. In this case, we know that jumping and wolf spiders have the largest supraesophageal ganglions (where learning and memory occur) of the arachnids ([116] in [117]), and that they operate in a heterogeneous environment because they primarily hunt other spiders, which have diverse behavioural routines and are patchily distributed. This gives us reason to expect them to exhibit behavioural flexibility underwritten by complex cognitive abilities. We also know that jumping spiders have excellent vision compared to other spiders [118] and that *Portia* is the most versatile spider genus in terms of its predatory behaviour: it hunts in the open, makes prey-capture webs, and, unique among spiders, it can use others' webs to hunt spider prey [119]. We also know something about the predatory behaviour of *P. fimbriata*: namely, that it waits until *Z. genicularis* is busy wrapping up an insect prey before moving across the web to attack it [118]. When *Z. genicularis* is wrapping its prey, it is less responsive to external movements on its web as well as to tactile stimulation, and *P. fimbriata* capitalizes on this unresponsiveness. Using vision to detect when *Z. genicularis* is wrapping prey, it times its approach with the prey wrapping behaviour, attacking the *Z. genicularis* when it is most vulnerable [118].

*Null hypothesis and burden of proof*: The traditional null hypothesis is insensitive to background evolutionary and ecological information and recommends selecting the simplest plausible hypothesis as the default. Here, this may mean assuming that *Z. genicularis* prey wrapping behaviour cues *P. fimbriata* to move (approach and attack) and that the association between prey wrapping behaviour and walking is nothing more than simple cue-recognition. However, this choice of null ignores predictions made by the ATM. Instead, we may propose a *contextual null hypothesis*, on which *P. fimbriata* tracks not simple cues but the attentional states of *Z. genicularis*, allowing it to time its movements with prey wrapping behaviour. Knowing when *Z. genicularis* is distracted would allow *P. fimbriata* to update its movement behaviour flexibly (e.g. detect and predict when the prey wrapping behaviour will finish and switch tactics if prey wrapping ends when *P. fimbriata* is in the middle of the web) and in different contexts (e.g. not only during prey wrapping behaviour). Because there is reason to suspect relatively complex forms of cognition in *P. fimbriata*, the contextual null hypothesis shifts the burden of proof onto the ‘simpler’ explanations, which may posit innate rules or learned behaviours that do not involve tracking attentional states of their prey.

*Testing the contextual null hypothesis*: The rationale behind selecting a contextual null hypothesis is that it is the best evidenced, and hence most likely to be true. For this reason, it is unnecessary to rule out alternatives to the contextual null even when these alternatives are simpler, though it does of course remain necessary to put the hypothesis to experimental test. In our example, one would need to test whether it is simply the onset and end of prey wrapping movements that initiate and terminate *P. fimbriata* movements. If initiation and termination of walking and prey wrapping are coordinated significantly more than expected by the contextual null, then this should decrease our confidence in the truth of the contextual null. On the other hand, if initiation and termination events are not significantly coordinated, then the contextual null hypothesis should be retained.

*Simplicity and the contextual null*: Although the contextual null hypothesis for the *P. fimbriata* in this example is arguably more complex than the traditional null hypothesis, the ATM may have issued a different recommendation had the background information been different. For instance, if *P. fimbriata* had been known to inhabit a homogeneous environment and had relatively small supraesophageal ganglions, the ATM would have recommended a simpler hypothesis, such as cue-recognition, as the null.

## 5. Conclusion

The convergent coevolution of flexible behaviours and higher brain architectures—and perhaps similar cognitive mechanisms—in distant phyla in response to broadly similar selection regimes, suggests that there may be a limited number of ways that nervous systems can be configured so as to produce flexible behaviour as a means for coping with heterogeneous, informationally demanding selective environments [104]. If the ATM proves to be robust, then it may serve as a theoretical buttress for the common and consequential assumption that flexible behaviour is evidence of complex cognition, while helping to overcome underdetermination problems and *a priori* simplicity preferences in comparative cognition in a way that is conducive to knowledge production. Establishing the presence of complex cognitive mechanisms in phylogenetically diverse lineages requires that we look beyond controlled experiment—and even beyond behavioural data—to draw upon a more diverse set of scientific methods and evidential sources. In short, it requires bringing the field of comparative cognition and its underlying subject matter—cognition itself—further under the ecological and evolutionary umbrella of biology.

## Endnotes

1 Although Shettleworth's account of cognition posits domain-specific cognitive architecture, there is nothing inherent to an information processing account that requires domain specificity; to the contrary, such accounts are consistent with some types of cognition involving domain-general information processing.

2 ‘Phenotypic plasticity’ refers to the developmental modifiability of organismic traits in response to environmental inputs. For morphology, developmental sensitivity to environmental inputs is generally limited to earlier phases of ontogeny and has largely irreversible effects. By contrast, behaviours can retain their flexibility throughout ontogeny. As West-Eberhard [19, p. 30] puts it, there is more potential for ontogenetic reorganization of behavioural subunits than there is for morphological or physiological ones.

3 This view broadly maps on to Godfrey-Smith's [21] useful distinction between ‘first-order plasticity’, which he defines as changeability in the organism's state, and ‘second-order plasticity’, which he defines as changeability of ‘the rules or conditionals that govern the organism's changes of state’.

4 For example, subordinate chimpanzees competing with dominants for food flexibly adjust their behaviour according to the knowledge state of the dominant [22]. Subordinates preferred food items that the dominant had not seen placed in the experimental room during baiting, as well as items that were moved after the dominant saw their initial placement. Subordinates were also sensitive to which dominant watched the baiting process: if the knowledgeable dominant was replaced with an uninformed dominant, subordinates preferred to take food items in front of the uninformed rather than the informed dominant. Chimpanzees showed behavioural flexibility because they learned to modify their behaviour in a new context based on their previous experience with hierarchical relationships in the group. This demonstration of behavioural flexibility and others like it have been taken as evidence of complex cognition [2].

5 The account of behavioural flexibility in this paper differs from commonly used species-level proxies of flexibility, such as innovations (the number of anecdotally novel foraging techniques used per species; e.g. [23]) and generalist versus specialist foraging strategies. These proxies are measured at the species level, whereas behavioural flexibility as understood here is measured at the individual level.

6 For a review of the many of meanings of Morgan's Canon, see Thomas [35]; for analyses and critiques of specific interpretations, see [36–39].

7 We suspect that the unduly strong emphasis on simplicity in comparative cognition is a vestige of the initial psychological rather than biological orientation of the field—for as Godfrey-Smith [51] notes, the preference for the Occamist null hypothesis is endemic in and largely peculiar to psychological science.

8 Note that when Godfrey-Smith refers to ‘cognition’, he may have in mind a more restrictive account of information processing than the big-tent account of cognition presupposes, as he uses the term ‘quasi-cognitive’ to describe non-mental information processing in organisms [61].

9 Although we expect neural structures to robustly correlate with other traits in the cluster, many different types of neural architecture may give rise to cognitive complexity [67], and thus the regularity will break down if our descriptions of the neural correlates of cognitive complexity are either too fine-grained or too broad. This explains why increased absolute or relative brain size is not an adequate basis by which to infer the existence of cognitive complexity in the context of observations of behavioural flexibility. In particular, it explains why an evolutionary increase in size or connectivity of any given brain region is neither necessary nor sufficient for inferring the evolution of cognitive complexity. It is not sufficient because although an observation of neuroanatomical growth or complexification does increase the subjective probability of cognitive complexity, there are many possible selective functions reflected by such changes that involve less complex forms of information processing, such as enhanced perception capabilities. It is not necessary because cognitive complexity could arise through the repurposing of existing brain structures without any measurable increase in neural tissue mass [68]. Without the additional observations of behavioural flexibility, a heterogeneous selective environment, and more fine-grained analyses of brain changes, the inference from neural complexification to cognitive complexity will be weak.

10 While current empirical support for the social brain hypothesis is correlational rather than directly causal, the absence of causal data does not necessarily mean that the hypothesis is under-evidenced. Correlational data may be the best evidence we can expect to find given the tandem coevolution of neurological and social complexity. Just as increasingly complex motor capacities co-evolve with increasingly complex perceptual capacities, complex social arrangements may evolve in tandem with increasingly complex brains, and the causal evolutionary interactions between them may be too fine-grained to resolve.

11 For instance, volumetric comparisons of the brain are difficult to interpret as enlargement of a brain region does not necessarily correlate with an increase in the number of neurons, and neuronal density may decrease as brains grow larger as has been found for some mammals and birds [113,114].
